# Childhood trauma and adult interpersonal relationship problems in patients with depression and anxiety disorders

**DOI:** 10.1186/s12991-014-0026-y

**Published:** 2014-09-16

**Authors:** Hyu Jung Huh, Sun-Young Kim, Jeong Jin Yu, Jeong-Ho Chae

**Affiliations:** 1 Department of Psychiatry, College of Medicine, Seoul St. Mary’s Hospital, The Catholic University of Korea, 222 Banpodaero, Seocho-Gu 137-701, Seoul, Republic of Korea; 2 Laboratory of Emotion, Catholic Institute of Medical Science and Biolife Industry, Seoul, Republic of Korea; 3 Faculty of Education, University of Tasmania, Hobart, Australia

**Keywords:** Childhood trauma, Interpersonal relationship, Depression, Anxiety

## Abstract

**Introduction:**

Although a plethora of studies have delineated the relationship between childhood trauma and onset, symptom severity, and course of depression and anxiety disorders, there has been little evidence that childhood trauma may lead to interpersonal problems among adult patients with depression and anxiety disorders. Given the lack of prior research in this area, we aimed to investigate characteristics of interpersonal problems in adult patients who had suffered various types of abuse and neglect in childhood.

**Methods:**

A total of 325 outpatients diagnosed with depression and anxiety disorders completed questionnaires on socio-demographic variables, different forms of childhood trauma, and current interpersonal problems. The Childhood Trauma Questionnaire (CTQ) was used to measure five different forms of childhood trauma (emotional abuse, emotional neglect, physical abuse, physical neglect, and sexual abuse) and the short form of the Korean-Inventory of Interpersonal Problems Circumplex Scale (KIIP-SC) was used to assess current interpersonal problems. We dichotomized patients into two groups (abused and non-abused groups) based on CTQ score and investigated the relationship of five different types of childhood trauma and interpersonal problems in adult patients with depression and anxiety disorders using multiple regression analysis.

**Result:**

Different types of childhood abuse and neglect appeared to have a significant influence on distinct symptom dimensions such as depression, state-trait anxiety, and anxiety sensitivity. In the final regression model, emotional abuse, emotional neglect, and sexual abuse during childhood were significantly associated with general interpersonal distress and several specific areas of interpersonal problems in adulthood. No association was found between childhood physical neglect and current general interpersonal distress.

**Conclusion:**

Childhood emotional trauma has more influence on interpersonal problems in adult patients with depression and anxiety disorders than childhood physical trauma. A history of childhood physical abuse is related to dominant interpersonal patterns rather than submissive interpersonal patterns in adulthood. These findings provide preliminary evidence that childhood trauma might substantially contribute to interpersonal problems in adulthood.

## 1 Background

Many studies over the last 20 years have shown that childhood trauma is related to the onset, symptom severity, and course of depression and anxiety disorders [1]-[3]. Various types of childhood trauma have been demonstrated to be associated with anxiety and depressive symptom severity [4],[5]. Although relationship between specific psychopathology such as depression and anxiety and different kinds of childhood traumatic events is still unclear, specific pathways from childhood trauma to psychopathology have been proposed in some theory. In cognitive-behavioral approach, the type of emotion is influenced by thought and belief contents activated by various types of childhood experience [6]. For example, depression is considered to be characterized by loss and self-deprecation, whereas anxiety is related with threat and danger [7],[8]. Thus, specific types of childhood trauma might cause specific symptoms in an individual with specific vulnerabilities [5].

In spite of a considerable body of literature on the relationship between childhood trauma and the various symptoms of depression and anxiety disorders, there were too few studies which investigated how childhood trauma affects on interpersonal problems in adults. Freud suggests that relationship schemas or patterns can be defined as organized representations of past behaviors and previous experiences in relationships [9],[10]. On the perspective of cognitive-behavioral theory, various types of maladaptive schematic representations of the self, world, and future are activated by matching specific life experience [5]. Specific types of maladaptive schema might influence specific adult attachment style and interpersonal relationships [11]. Although these theories have a deep impact on many different types of current psychotherapy, there were few clinical studies to support this position. Recently, a few studies have suggested that survivors of childhood trauma may experience lower relationship quality, intimacy dysfunction, and social adjustment difficulties [12],[13]. While most research has focused on childhood sexual trauma in community samples [14]-[16], relatively few studies have attempted to clarify the impact of various kinds of trauma such as emotional or physical trauma on interpersonal problems in clinically ill patients [17].

Much evidence suggests that relatively worse pretreatment social functioning is associated with earlier age of onset, higher levels of depressive symptoms, and lower remission rates after treatment [18]-[22]. Furthermore, there is a lot of evidence that childhood trauma is a risk factor for more frequent recurrent episode, chronicity of depression, and suicidality [8],[23]-[27]. Therefore, it is crucial to investigate the relationship between childhood traumatic experiences and adult interpersonal and social functioning in order to find one of the mediating factors between childhood trauma experiences and treatment outcome in depression and anxiety disorders [28].

Given the paucity of research, the present study aims to investigate the association between different types of childhood trauma and interpersonal problem in adult patient with depression and anxiety disorders. Furthermore, the present study seeks to clarify the specific interpersonal pattern in different types of childhood trauma and neglect in patients with depression and anxiety disorders.

## 2 Methods

### 2.1 Participant

During the 12-month study period between August 2012 and July 2013, patients who firstly visited Mood and Anxiety Disorders Unit at Seoul St. Mary's Hospital, The Catholic University of Korea, and met the DSM-IV diagnostic criteria for depressive and/or anxiety disorders were recruited consecutively. Diagnosis was conducted by a psychiatrist (JHC) using semi-structured diagnostic interviews of the Mini-International Neuropsychiatric Interview (M.I.N.I.) [29]. Eligibility criteria included being 18–65 years of age and literate in Korean. Exclusion criteria included a lifetime diagnosis of psychotic disorder, bipolar disorder, mental retardation, and any mental disorder due to a general medical condition. We also excluded individuals with significant personality disorders and/or medical problems that would interfere with study participation. A total of 351 outpatients who met the inclusion and exclusion criteria consented to participate in this study. Restricting analyses to those who had completed all measures, the final sample included 325 patients. The study procedure was approved by the institutional review boards of the ethical committee of the Seoul St. Mary's Hospital at the Catholic University of Korea. This study was in compliance with the Helsinki Declaration. Written informed consent was obtained from the patient for the publication of this report and any accompanying images.

### 2.2 Instruments

#### 2.2.1 Demographics and psychiatric symptoms

During clinical interviews, we asked patients about demographic information such as years of formal education, marital status, and employment status.

Among the psychiatric symptoms, we assessed the participants' symptoms of depression, anxiety, and anxiety sensitivity using the Beck Depression Inventory (BDI) [30], the State-Trait Anxiety Inventory (STAI) [31], and Anxiety Sensitivity Index-Revised (ASI-R) [32],[33]. Korean versions of BDI [34], STAI [35], and ASI-R [36] were all well validated.

#### 2.2.2 Childhood trauma

Childhood abuse and neglect was assessed using the Childhood Trauma Questionnaire (CTQ) [37], a 28-item self-report inventory assessing five types of trauma experienced as a child and a teenager: emotional, physical, and sexual abuse and emotional and physical neglect. Items are rated on a 5-point frequency scale (1 = never true to 5 = very often true) and summed to yield a total score for each trauma, ranging from 5 to 25, with higher scores indicative of greater severity. The CTQ provides three thresholds/cut-score (mild, moderate, and severe) for each type of trauma. To minimize false identification of trauma, moderate thresholds (>12 for emotional abuse, >9 for physical abuse, >7 for sexual abuse, and >14 and >9 for emotional and physical neglect, respectively) were used to dichotomize all scores (abused vs non-abused or neglected vs non-neglected) for descriptive purposes. We also defined emotional trauma (emotional abuse and emotional neglect) and physical trauma (physical abuse and physical neglect) as >21 emotional trauma and >18 physical trauma. Therefore, we used operational definition as non-abused vs abused or non-neglected vs neglected group according to the classification of CTQ scale scores. Korean version of CTQ was also validated [38].

#### 2.2.3 Adulthood interpersonal problems

Current interpersonal problems were assessed using the short form of the Korean-Inventory of Interpersonal Problems Circumplex Scale (KIIP-SC), a 40-item self-report inventory assessing eight dimensions of interpersonal problems. The respondents were asked to indicate to what degree he or she experiences a set of 40 different behaviors as difficult or done too much on a 5-point scale (‘not at all’, ‘a little’, ‘moderately’, ‘quite a lot’, ‘a lot’).

**Figure 1:**
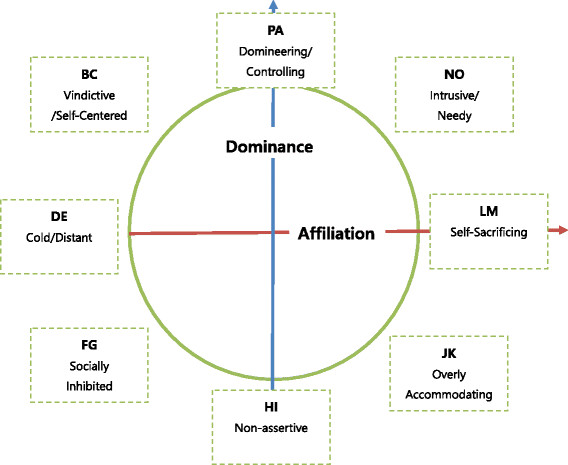
Inventory of Interpersonal problems circumplex subscales (Hardy AO et al, 2011).

The measure is based on a theoretical circumplex structure of interpersonal behavior and has received considerable research support on its structural validity [39]-[41]. It yields global interpersonal distress, two dimensional scores of dominance distress and affiliation distress, and octant scores which indicate eight dimensions of interpersonal problems constituting a circumplex of personality: domineering/controlling (PA), vindictive/self-centered (BC), cold/distant(DE), socially inhibited (FG), nonassertive (HI), overly accommodating (JK), self-sacrificing (LM), and intrusive/needy (NO) [42]. Figure 1 describes the circumplex structure of Inventory of Interpersonal Problems.

To analyze the specific pattern of interpersonal problems associated with childhood abuse and neglect, the following formulas were used to calculate affiliation, dominance, and length scores [43].

(1) $$\begin{array}{l} \mathrm{Affiliation}\;=\mathrm{LM}+0.71\left(\mathrm{NO}+\mathrm{JK}\right)-0.71\left(\mathrm{BC}+\mathrm{FG}\right)-\mathrm{DE}\\ \mathrm{Dominance}=\mathrm{PA}+0.71\left(\mathrm{BC}+\mathrm{NO}\right)-0.71\left(\mathrm{FG}+\mathrm{JK}\right)-\mathrm{HI}\\ \mathrm{Length}\;=\sqrt{\left(\mathrm{Affiliatio}n^{2}+\mathrm{Dominanc}e^{2}\right)} \end{array}$$

The affiliation score represents behavioral dimensions ranging from friendly to hostile, while the dominance score means behavioral patterns ranging from submissive to dominant attitudes in interpersonal relationships. The length score is an indication of how exclusively one experiences a specific interpersonal problem and also reflects interpersonal rigidity [43],[44]. The Korean version of Inventory of the Interpersonal Problems Circumplex Scale (KIIP-SC) was also validated [45].

### 2.3 Statistical analysis

All statistical analyses were performed using the Statistical Package for Social Sciences (SPSS) version 18.0 (SPSS Inc., Chicago, USA). To compare demographic and clinical characteristics of the groups with and without childhood trauma, we performed independent *t* tests for continuous variable and *x*^2^ tests for categorical variables.

Stepwise multiple regression analysis with backward elimination was used to examine the effect of childhood abuse and neglect on interpersonal problems in adulthood after controlling for age, educational level, scores of BDI and ASI-R, and other variables that were significant in the bivariate analysis.

Since we consider the possibility that the more depressive or anxious patients tend to report greater interpersonal problems due to cognitive distortions, we controlled for BDI, ASI-R, and Trait Anxiety Inventory scores as covariates. Separate analyses were conducted for the KIIP-SC total score, each eight dimensional subscore, KIIP-SC affiliation, dominance, and length, which served as dependent variables. The experience of childhood trauma and neglect served as an independent variable.

In addition, we divided patients as non-traumatized group, only emotional traumatized group, only physical traumatized group, and both emotional and physical traumatized group to analyze co-occurrence effect of childhood trauma. ANCOVA (analysis of covariance) was used to compare four groups, controlling covariate as BDI, ASI, and STAI. All results were considered significant at *p* = 0.05, two-tailed.

## 3 Results

A total of 325 participating patients had the following principal diagnosis: 174 (53.5%) patients had depressive disorders comprising major depressive disorder (*N* = 121), dysthymic disorder (*N* = 3), and depressive disorder not otherwise specified (NOS) (*N* = 50). The remaining 151 (46.5%) patients had anxiety disorders comprising panic disorder (*N* = 40), generalized anxiety disorder (*N* = 30), obsessive compulsive disorder (*N* = 20), posttraumatic stress disorder (*N* = 18), social anxiety disorder (*N* = 8), and anxiety disorder NOS (*N* = 35). Among them, 43 (13.7%) were diagnosed as having both depressive and anxiety disorders. The mean (±SD) KIIP-SC score in all patients was 65.07 (±25.78).

Patients with depressive disorder reported more childhood emotional abuse (30.1% vs 14.4%, *p* = 0.001) and physical abuse (45.1% vs 26.7%, *p* < 0.001) and childhood emotional neglect (82.9% vs 73.6%, *p* = 0.048) than those with anxiety disorders, while there were no significant differences in physical neglect (80.6% vs 82.3%, *p* = 0.781) and sexual abuse in childhood (20.6% vs 15.6%, *p* = 0.259) between patients with depressive disorder and those with anxiety disorder. Patients with depressive disorder had significantly higher KIIP-SC scores (69.40 ± 24.02) than those with anxiety disorder (60.90 ± 26.79) (*p* = 0.003).

### 3.1 Demographic and clinical characteristics of the groups with and without childhood trauma in adult patients with depression and anxiety disorders

Table 1 summarizes demographic and clinical characteristics of the groups with and without trauma in patients with depression and anxiety disorders. Younger patients reported higher levels of emotional abuse (33.73 ± 12.47 vs 38.13 ± 13.28, *p* = 0.011) and physical neglect (36.26 ± 12.54 vs 40.69 ± 15.30, *p* = 0.039) than older patients. Patients without a history of childhood emotional abuse were more likely to have an intact marriage than those with a history of childhood emotional abuse (27.1% vs 48.8%, *p* = 0.002). Lower levels of formal education (4.81 ± 1.38 vs 5.19 ± 1.30, *p* = 0.049) and lower unemployment rates (51.5% vs 35.1%, *p* = 0.028) were found among patients with a history of childhood physical neglect than those without such a history.

**Table 1:** Demographic and clinical characteristics of the groups with childhood trauma and without childhood trauma in patients with depression and anxiety disorders

	**Emotional abuse**		**Emotional neglect**		**Physical abuse**		**Physical neglect**		**Sexual abuse**	
	**Yes (*n*** **= 70)**	**No (*n*** **= 255)**	**Yes (*n*** **= 255)**	**No (*n*** **= 70)**	**Yes (*n*** **= 115)**	**No (*n*** **= 210)**	**Yes (*n*** **= 60)**	**No (*n*** **= 265)**	**Yes (*n*** **= 60)**	**No (*n*** **= 265)**
*Age*	33.73 ± 12.47	38.13 ± 13.28	36.67 ± 12.79	38.94 ± 14.34	35.65 ± 12.44	37.78 ± 13.51	36.26 ± 12.54	40.69 ± 15.30	39.92 ± 12.79	36.35 ± 13.22
	*0.011*		0.239		0.155		*0.039*		0.056	
*Gender (female)*	60.3%	39.7%	54.0%	59.2%	48.3%	57.9%	54.8%	51.6%	55.7%	53.9%
	0.351		0.502		0.109		0.674		0.887	
*Educational years*	4.86 ± 1.40	5.19 ± 1.30	5.06 ± 1.34	5.27 ± 1.29	5.18 ± 1.27	5.09 ± 1.34	5.19 ± 1.30	4.81 ± 1.38	4.90 ± 1.27	5.15 ± 1.34
	0.080		0.240		0.559		*0.049*		0.178	
*Marital status (yes)*	27.1%	48.8%	43.7%	46.2%	37.8%	48.0%	42.7%	45.8%	39.0%	44.6%
	*0.002*		0.780		0.082		0.771		0.469	
*Employment status (yes)*	50.0%	48.1%	48.0%	50.0%	54.8%	45.3%	51.5%	35.1%	41.8%	50.4%
	0.788		0.785		0.128		*0.028*		0.299	
*Depression (BDI)*	28.57 ± 11.60	22.76 ± 12.06	25.57 ± 12.08	18.90 ± 11.16	27.75 ± 11.78	21.95 ± 11.92	24.17 ± 12.21	23.50 ± 11.91	29.07 ± 12.26	22.90 ± 11.87
	*0.000*		*0.000*		*0.000*		0.692		*0.001*	
*State anxiety (SAI)*	60.73 ± 11.73	56.43 ± 13.07	58.86 ± 12.01	52.45 ± 14.51	60.25 ± 11.42	55.60 ± 13.43	57.78 ± 12.88	56.74 ± 13.30	61.33 ± 12.05	56.05 ± 13.04
	*0.008*		*0.001*		*0.001*		0.336		*0.007*	
*Trait anxiety (TAI)*	63.56 ± 10.42	57.49 ± 12.47	60.17 ± 11.83	54.01 ± 12.72	61.69 ± 10.45	57.08 ± 12.88	59.41 ± 11.89	56.74 ± 13.30	61.80 ± 10.91	58.17 ± 12.49
	*0.000*		*0.000*		*0.000*		0.152		*0.026*	
*Anxiety-sensitivity (ASI-R)*	98.13 ± 34.43	88.77 ± 34.47	92.38 ± 34.23	85.86 ± 37.20	100.13 ± 32.18	86.13 ± 35.64	92.68 ± 34.79	82.16 ± 33.10	104.23 ± 33.85	87.73 ± 34.18
	*0.045*		0.182		*0.000*		*0.029*		*0.001*	

BDI scores were significantly higher in patients with a history of childhood emotional abuse (28.57 ± 11.60 vs 22.76 ± 12.06, *p* < 0.001), emotional neglect (25.57 ± 12.08 vs 18.90 ± 11.16, *p* < 0.001), physical abuse (27.75 ± 11.78 vs 21.95 ± 11.92, *p* < 0.001), and sexual abuse (29.07 ± 12.26 vs 22.90 ± 11.87, *p* < 0.001) than in those without such a history. Patients with a history of childhood emotional abuse (60.73 ± 11.73 vs 56.43 ± 13.07. *p* = 0.008), emotional neglect (58.86 ± 12.01 vs 52.45 ± 14.51, *p* = 0.001), physical abuse (60.25.86 ± 11.42 vs 55.60 ± 13.43, *p* < 0.001), and sexual abuse (61.33.86 ± 12.05 vs 56.05 ± 13.04, *p* = 0.007) had higher state anxiety scores than those without such as history. Trait anxiety scores were also significantly higher in patients with a history of childhood emotional abuse (63.56 ± 10.42 vs 57.49 ± 12.47, *p* < 0.001), emotional neglect (60.17 ± 11.83 vs 54.01 ± 12.72, *p* < 0.001), physical abuse (61.69 ± 10.45 vs 57.08 ± 12.88, *p* < 0.001), and sexual abuse (61.80 ± 10.91 vs 58.17 ± 12.49, *p* = 0.026) than in those without such a history. Similarly, patients with a history of childhood emotional abuse (98.13 ± 34.43 vs 88.77 ± 34.47, *p* = 0.045), physical abuse (100.13 ± 32.18 vs 86.45 ± 35.55, *p* < 0.001), physical neglect (92.93 ± 34.67 vs 82.16 ± 33.10, *p* = 0.029), and sexual abuse (104.23 ± 33.85 vs 87.98 ± 34.09, *p* < 0.001) had higher ASI-R scores than those without such a history.

### 3.2 Effect of childhood abuse and neglect on interpersonal problems in adulthood

Table 2 shows the associations between different types of childhood trauma and KIIP-SC total score, two dimensional scores of dominance distress and affiliation distress, and octant scores after controlling for BDI, ASI-R, and Trait Anxiety Inventory scores and other demographic variables that were significantly related to a history of childhood trauma.

**Table 2:** Effect of childhood abuse and neglect associated on adult interpersonal problems (adjusted by demographics and psychiatric symptoms)

	**Emotional abuse**	**Emotional neglect**	**Physical abuse**	**Physical neglect**	**Sexual abuse**
	**β(*p*** **value)**	**β(*p*** **value)**	**β(*p*** **value)**	**β(*p*** **value)**	**β(*p*** **value)**
KIIP-SC	*0.108(0.041)*	*0.178(0.001)*	0.061(0.249)	0.033(0.528)	*0.133(0.010)*
Domineering/controlling (PA)	*0.232(0.000)*	*0.211(0.000)*	*0.213(0.000)*	0.016(0.795)	*0.278(0.000)*
Vindictive/self-centered (BC)	0.050(0.414)	0.106(0.075)	0.059(0.324)	−0.023(0.700)	0.071(0.234)
Cold/distant (DE)	0.053(0.358)	0.071(0.210)	0.034(0.568)	−0.039(0.488)	0.002(0.975)
Socially inhibited (FG)	0.080(0.155)	0.102(0.069)	0.024(0.666)	0.058(0.294)	0.076(0.170)
Nonassertive (HI)	0.018(0.747)	*0.120(0.029)*	−0.089(0.105)	0.047(0.386)	−0.039(0.474)
Overly accommodating (JK)	0.099(0.071)	*0.160(0.003)*	0.008(0.882)	0.049(0.366)	*0.116(0.031)*
Self-sacrificing (LM)	0.082(0.136)	*0.107(0.050)*	0.023(0.679)	0.053(0.335)	*0.181(0.001)*
Intrusive/needy (NO)	*0.133(0.022)*	*0.159(0.006)*	*0.120(0.038)*	0.022(0.705)	*0.214(0.000)*
Affiliation	0.038(0.548)	0.043(0.498)	0.004(0.946)	0.045(0.469)	0.116(0.059)
Dominance	0.084(0.162)	−0.014(0.819)	*0.164(0.006)*	−0.067(0.255)	*0.166(0.005)*
Length	−0.055(0.365)	0.078(0.194)	0.012(0.837)	0.027(0.646)	−0.073(0.223)

A history of childhood emotional abuse (*p* = 0.041), emotional neglect (*p* = 0.001), and sexual abuse (*p* = 0.010) appeared to be associated with the KIIP-SC total score, which represents general interpersonal problems. No significant associations were found between a history of childhood physical abuse and physical neglect and the KIIP-SC total score.

Considering the octant scores of the KIIP-SC, patients who had experienced childhood emotional abuse were more domineering/controlling (*p* < 0.001) and intrusive/needy (*p* = 0.022) than those who had not experienced such maltreatment. Patients with a childhood history of emotional neglect were more likely to be domineering/controlling (*p* < 0.001), nonassertive (*p* = 0.029), overly accommodating (*p* = 0.003), self-sacrificing (*p* = 0.050), and intrusive/needy (*p* = 0.006) than those with no such history.

Although no significant difference was found in the KIIP-SC total score between patients with a history of physical abuse in childhood and those without such a history, the former were more likely to be domineering/controlling (*p* = 0.001) and intrusive/needy (*p* = 0.038) than the latter. There were no significant differences in either the KIIP-SC total score or other subscores of KIIP-SC between patients with a history of childhood physical neglect and those without such a history.

In addition to general interpersonal distress, patients who had experienced sexual abuse in childhood were more likely to be domineering/controlling (*p* < 0.001), overly accommodating (*p* = 0.031), self-sacrificing (*p* = 0.001), and intrusive/needy (*p* = 0.013) than those without a history of childhood sexual abuse.

Affiliation distress scores were not related to any forms of childhood abuse and neglect. However, patients with a history of physical (*p* = 0.006) or sexual abuse in childhood (*p* = 0.005) were found to be in a more dominant position in interpersonal relationships than those with no history of childhood physical abuse.

### 3.3 Comparison of BDI, STAI, ASI-R, and adult interpersonal problems by groups with co-occurrence or without co-occurrence of childhood emotional and physical trauma

**Figure 2:**
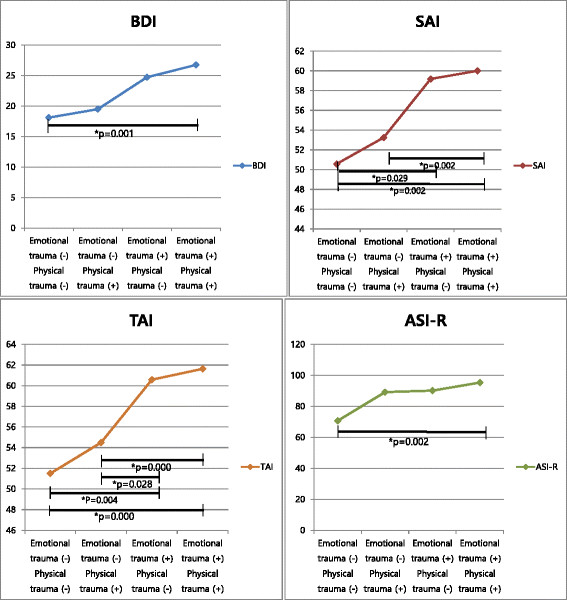
Comparison of BDI, STAI, and ASI-R by groups with co-occurrence or without co-occurrence of childhood emotional and physical trauma.

**Figure 3:**
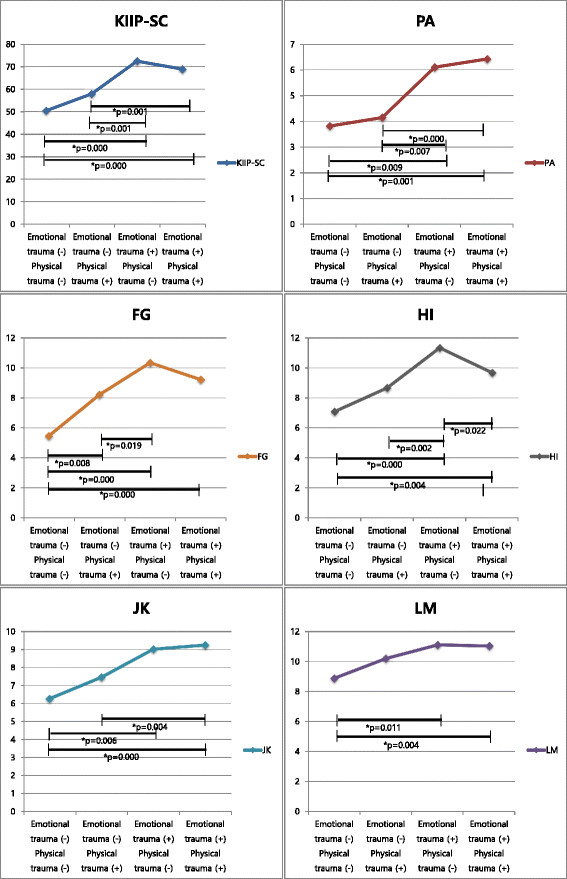
Comparison of adult interpersonal problems by groups with co-occurrence or without co-occurrence of childhood emotional and physical trauma (mean ± standard error adjusted by demographics and psychiatric symptoms).

Tables 3 and 4 and Figures 2 and 3 summarize comparison of BDI, STAI, ASI-R, and adult interpersonal problems by groups with co-occurrence or without co-occurrence of childhood emotional and physical trauma. All of the symptom severity scores were significantly higher in patients with both emotional and physical trauma than non-traumatized group (*p* = 0.001 for BDI, *p* = 0.002 for SAI, *p* < 0.001 for TAI, and *p* = 0.002 for ASI-R). Co-occurrence effect of emotional and physical trauma appeared only in state and trait anxiety. State anxiety and trait anxiety scores were significantly higher in patients with both emotional and physical trauma than patients with only physical trauma (*p* = 0.002 for SAI and *p* < 0.001 for TAI).

**Table 3:** Comparison of BDI, STAI, and ASI-R by groups with co-occurrence or without co-occurrence of childhood emotional and physical trauma

	**Emotional trauma**	**Physical trauma**	**Emotional trauma**	**Physical trauma**	**Emotional trauma**	**Physical trauma**	**Emotional trauma**	**Physical trauma**	***p*** **value**
	**No**	**No**	**No**	**Yes**	**Yes**	**No**	**Yes**	**Yes**	
	***n*** **= 32**		***n*** **= 73**		***n*** **= 50**		***n*** **= 170**		
Depression (BDI)	18.10 (±11.72)		19.49 (±11.93)		24.73 (±11.69)		26.74 (±11.65)		*0.000*
State anxiety (SAI)	50.58 (±13.05)		53.25 (±15.09)		59.18 (±11.78)		60.00 (±12.83)		*0.000*
Trait anxiety (TAI)	51.50 (±11.87)		54.49 (±12.91)		60.58 (±12.49)		61.63 (±10.83)		*0.000*
Anxiety-sensitivity (ASI-R)	70.73 (±28.56)		89.16 (±37.66)		90.14 (±35.90)		95.34 (±32.85)		*0.004*

**Table 4:** **Comparison of adult interpersonal problems by groups with co-occurrence or without co-occurrence of childhood emotional and physical trauma (mean ± standard error adjusted by demographics and psychiatric symptoms**)

	**Emotional trauma**	**Physical trauma**	**Emotional trauma**	**Physical trauma**	**Emotional trauma**	**Physical trauma**	**Emotional trauma**	**Physical trauma**	***p*** **value**
	**No**	**No**	**No**	**Yes**	**Yes**	**No**	**Yes**	**Yes**	
	***n*** **= 32**		***n*** **= 73**		***n*** **= 50**		***n*** **= 170**		
KIIP-SC	50.38 (4.60)		57.86 (2.76)		72.41 (3.24)		68.85 (1.70)		*0.000*
Domineering/controlling (PA)	3.81 (0.68)		4.14 (0.47)		6.10 (0.55)		6.42 (0.286)		*0.000*
Vindictive/self-centered (BC)	5.24 (0.84)		5.10 (0.57)		7.11 (0.67)		6.51 (0.35)		0.067
Cold/distant (DE)	6.65 (0.89)		6.84 (0.61)		8.95 (0.71)		8.23 (0.37)		0.059
Socially inhibited (FG)	5.46 (0.86)		8.21 (0.58)		10.34 (0.68)		9.21 (0.36)		*0.000*
Nonassertive (HI)	7.08 (0.80)		8.66 (0.54)		11.33 (0.64)		9.67 (0.36)		*0.000*
Overly accommodating (JK)	6.27 (0.77)		7.46 (0.52)		9.02 (0.61)		9.25 (0.32)		*0.001*
Self-sacrificing (LM)	8.88 (0.68)		10.20 (0.46)		11.11 (0.54)		11.04 (0.28)		*0.022*
Intrusive/needy (NO)	7.00 (0.72)		7.27 (0.49)		8.47 (0.57)		8.51 (0.30)		0.074
Affiliation	4.06 (2.10)		4.35 (1.43)		2.19 (1.68)		4.29 (0.88)		0.725
Dominance	−2.91 (1.74)		−6.84 (1.18)		−7.90 (1.39)		−5.69 (0.73)		0.120
Length	10.35 (1.43)		13.90 (0.97)		14.00 (1.14)		14.07 (0.60)		0.120

Significant difference has appeared in KIIP-SC (*p* < 0.001), dominant/controlling (*p* = 0.001), socially inhibited (*p* < 0.001), non-assertive (*p* = 0.004), overly accommodating (*p* < 0.001), and self-sacrificing scores (*p* = 0.004) between patients with history of both emotional and physical trauma and patients with no history of childhood trauma. Co-occurrence effect was observed in general interpersonal problem and dominant/controlling, non-assertive, and overly accommodating interpersonal pattern. KIIP-SC total score (*p* = 0.001), dominant/controlling (*p* < 0.001), and overly accommodating subscores (*p* = 0.004) were higher in patients with both childhood emotional and physical trauma than patients with only physical trauma. Non-assertive subscore (*p* = 0.022) was lower in patients with both childhood emotional and physical trauma than patients with only emotional trauma.

## 4 Discussion

Although childhood trauma has been extensively investigated on symptomatic perspectives in psychiatrically ill patients, little evidence has been provided whether interpersonal problems in adulthood originated from childhood abuse and neglect. Furthermore, most of the studies about the effect of childhood trauma on interpersonal relationships in adulthood have been done using community samples. To our best knowledge, there is only one study to investigate the characteristics of interpersonal patterns in 119 psychiatrically ill adult patients who experienced various types of childhood trauma [17]. In feeling this void, the present study examines the relationships between different types of childhood trauma and adult interpersonal problems in a relatively large clinical sample. Below, we summarize our findings and discuss their limitations and implications.

First of all, from a symptomatic perspective, depressive symptoms, state-trait anxiety, and anxiety sensitivity are related to various types of childhood trauma, although a specific symptom dimension, especially anxiety sensitivity, has different relationships with different types of childhood trauma.

Based on the accumulated evidence, it is reasonable to assume that childhood trauma is an important risk factor for depression and anxiety disorders in adulthood [3],[46],[47]. Adding to prior work, our findings show that patients with a history of childhood trauma report significantly higher depression and anxiety symptom severity than those without these experiences. In addition to the symptom severity of depression and anxiety, childhood trauma is known to be related with other important psychopathology such as dissociation, affecting age of onset, chronicity, and recurrence of anxiety/depressive disorder. For this reason, several researchers have delineated new concepts about trauma-related syndrome like ‘dissociative depression’ [48],[49].

Anxiety sensitivity was significantly associated with childhood physical neglect but not with childhood emotional neglect, whereas depressive symptom and state-trait anxiety were not significantly associated with childhood physical neglect. There are at least two possible interpretations of this finding. First, each type of childhood trauma is associated with a different component (e.g., physical, cognitive, behavioral) of anxiety and depression. Previous factor analytic studies indicate that Anxiety Sensitivity Index (ASI) is comprised of three lower-order components representing physical, psychological, and social concerns [32],[50],[51]. In contrast, the item content of the State-Trait Anxiety Inventory (STAI) primarily refers to cognitive symptoms of anxiety [52].

Another possibility is that childhood trauma is differently associated with specific symptom dimensions of depression and anxiety. A series of factor analyses to evaluate the STAI-T (trait anxiety) found that it is comprised of both an anxiety factor and a depression factor, but not specific for anxiety [51],[53]. Previous research has found that emotional neglect and psychological abuse during childhood have a stronger association with pure depression than pure anxiety, whereas physical trauma during childhood was associated only with anxious arousal [5]. Consistent with prior research, childhood emotional neglect is significantly related to BDI and STAI scores, but not to ASI scores. Furthermore, childhood physical abuse and neglect is significantly associated with anxiety sensitivity.

Another important finding in this study is that childhood abuse and neglect, especially emotional abuse, emotional neglect, and sexual abuse, contributes to interpersonal problems in adult patients with depression and anxiety disorders. Patients with a history of emotional abuse, neglect, or sexual abuse in childhood report more general interpersonal distress than those without such a history. Although patients with a history of childhood physical abuse do not report higher levels of general interpersonal distress, they report significantly higher levels of dominant/controlling and intrusive/needy interpersonal patterns than non-abused patients. However, childhood physical neglect is not associated with any areas of interpersonal difficulty.

Extensive evidence suggests that childhood abuse can lead to difficulty with intimate relationships later in life and the formation of a secure attachment [12],[13],[47],[54],[55]. Thus, it can be inferred that childhood trauma leads to problems with interpersonal relationships in adult patients. However, little has been demonstrated with a clinical sample of psychiatrically ill patients about the impact of the childhood trauma on interpersonal functioning in adulthood. Our study thus represents an important addition to the literature.

Although the underlying mechanisms between childhood trauma, specific symptoms, and interpersonal problems are unclear, biological effect of trauma is the disturbance of the stress response systems, including the HPA axis and the CRF system. Increasing stress sensitivity would lower the threshold to provoke depression and anxiety [56]. Regarding interpersonal relationship, childhood trauma can disrupt the development of attachment to others and reflective awareness of self and others [25]. It might lead to difficulty with interpersonal functioning. Furthermore, clinical characteristics such as earlier age of onset, chronicity, and more recurrent episodes of trauma-related depression might interrupt the interpersonal relationship because patients with trauma-related depression suffered from illness for a longer period of their life [8],[23]-[26].

Third, all types of childhood trauma except physical neglect have a significant influence on dominant/controlling and intrusive/needy interpersonal patterns. Items assessing dominant/controlling and intrusive/needy patterns are related to aggressiveness; thus, these findings implicate that victims of childhood trauma are at risk for abusive or traumatic relationships in adulthood. In other words, patients who had experienced childhood trauma may attempt to resolve the emotional turmoil associated with traumatic events by organizing their interpersonal relationships in a way that allows some degree of perceived control and attempt to develop a sense of mastery by being the initiator of the potentially traumatic interactions [9],[10],[57]. It remains for future research to inquire directly about the link between childhood trauma and later aggression and delinquency [58]-[60].

Fourth, our findings suggest that childhood sexual abuse is more strongly associated with diverse interpersonal problems in adulthood than other abusive trauma such as emotional and physical trauma. Patients who had experienced sexual abuse in childhood suffered from domineering/controlling, overly accommodating, self-sacrificing, and intrusive/needy interpersonal patterns. Therefore, childhood sexual abuse seems to have contrasting interpersonal patterns such as dominant and submissive attitude simultaneously. There are at least two possible interpretations of this finding.

One possibility is that childhood sexual abuse may involve both emotional and physical trauma. That is, childhood sexual trauma is largely associated with adverse psychological, behavioral, and social consequences.

Another interpretation is that sexually abused victims tend to show some ambivalence about wanting to be close enough to others to obtain help but not wanting to be hurt [12],[14],[17]. Thus, our findings may reflect sexually abused victims' ambivalent feelings toward others and dominant or submissive attitude in interpersonal relations.

Fifth, we found that patients who had experienced childhood emotional neglect exhibited a wide range of interpersonal problems like those with a history of childhood sexual abuse. In addition to domineering/controlling and intrusive/needy interpersonal problems, these patients reported nonassertive, overly accommodating, and self-sacrificing interpersonal problems. Indeed, there is some evidence to suggest that a history of childhood neglect, either physical or emotional, is related to a lower prevalence of the wish for self-assertion [17].

In a similar vein, previous research has suggested that childhood emotional neglect is closely related to later anhedonic depression [5]. Therefore, it might be possible that participants reporting childhood emotional neglect tended to interpret their interpersonal situations negatively. Future studies would be needed to further elaborate on the findings of this study.

Finally, no association was found between childhood physical neglect and adult interpersonal relationship problems. Our finding is inconsistent with previous studies showing that patients with a history of childhood physical neglect are more likely to seek comfort and to have difficulty in self-assertion [17],[61]. Because our results did not consider a dose-response relationship between childhood trauma and interpersonal relationships in adulthood, more research efforts would be required to address the relationship between childhood physical neglect and later interpersonal relationships in more detail.

Sixth, considering co-occurrence of various type of childhood trauma, trait anxiety and state anxiety tend to be more severe in patients with co-occurrence of emotional and physical trauma than patients with only physical trauma. Regarding adult interpersonal problem, general interpersonal distress and dominant/controlling and overly accommodating interpersonal pattern were more frequent in patients with co-occurrence of emotional and physical trauma than patients reporting only physical trauma. On the other hand, non-assertive interpersonal problem subscore appeared to be lower in patients with both types of childhood trauma than patients with only emotional trauma. A lot of previous research suggested that childhood physical abuse is related with interpersonal aggression or violent behavior [62],[63]. Furthermore, there is a possibility that patients with both physical and emotional trauma inherit genes related with aggressive behavior from their parents [64],[65]. Therefore, it might be possible that patients with both types of trauma have more assertive attitude than patients with only emotional trauma by gene-environment interactions [66].

Several limitations are needed to be considered in the present study. First, although widely used and well validated, CTQ and KIIP-SC are self-report measures which may not always provide accurate information. Although depressive and anxiety symptoms were used as control variables in the present study, it is possible that many patients might have distorted mental representations on their interpersonal problems and traumatic memory due to depressive or anxiety symptoms. Moreover, recall reports might be biased because of age differences between the childhood trauma and the non-trauma groups. Second, because we did not analyze separately anxiety and depressive patients, it is possible that distinct patterns of interpersonal problems might exist depending on different symptom dimensions such as depression and anxiety. Further research would need to elaborate on the distinctive interpersonal patterns between anxiety and depressive disorder. Third, although childhood trauma is also known to be related with many other psychiatric conditions such as dissociative disorders and borderline personality, we could not consider the possibility of comorbidity with these psychiatric conditions and anxiety/depressive disorder in detail [48],[49]. Future study would be essential to delineate more elaborative clinical characteristics such as existence of dissociation and personality disorder, earlier age of onset, chronicity, and more frequent recurrence of anxiety/depressive disorder related with childhood trauma.

In spite of several limitations, this study represents an important attempt to demonstrate the effect of childhood trauma on interpersonal relationships in adulthood in a relatively large sample of psychiatric patients. Our findings about the relationship among childhood trauma, specific symptoms, and adult interpersonal relationship problems provide basic evidence that different and individualized psychological intervention would be required according to specific types of childhood trauma.

## 5 Conclusion

In this study, we examined the relationship between childhood trauma and later adult interpersonal problems. Different types of childhood abuse and neglect appeared to influence specific symptom dimensions and interpersonal relationship in adulthood. Especially emotional abuse, emotional neglect, and sexual abuse in childhood contributed more to diverse interpersonal problems in adulthood than did physical abuse and neglect in childhood. The present study provides preliminary evidence that childhood trauma has an adverse impact on interpersonal problems in adulthood. More elaborate studies to delineate the relationship between childhood trauma and later interpersonal relationships would be required in the future.

## Competing interests

This research was supported by a grant from the Korea Research Foundation, NRF-2012R1A1B3001314 and NRF-2006-2005152. The Korea Research Foundation did not play further role in study design; in the collection, analysis, and interpretation of data; in writing of the manuscript; or in the decision to submit the paper for publication. Dr. J-H Chae has received research grant from the Korea Research Foundation. All other authors declare that they have no conflict of interest.

## Authors' contributions

HHJ performed the study by design of the study, analysis of data, and writing of the papers. SYK contributed to data collection. JJY contributed to theoretical interpretation and provided significant input on the manuscript. JHC contributed to design of the study, supervised the data collection, and provided significant input on the manuscript. All authors read and approved the final manuscript.
